# Nutrient Sensing Systems in Fish: Impact on Food Intake Regulation and Energy Homeostasis

**DOI:** 10.3389/fnins.2016.00603

**Published:** 2017-01-05

**Authors:** Marta Conde-Sieira, José L. Soengas

**Affiliations:** Laboratorio de Fisioloxía Animal, Departamento de Bioloxía Funcional e Ciencias da Saúde, Facultade de Bioloxía, Universidade de VigoVigo, Spain

**Keywords:** nutrient sensors, fish, hypothalamus, liver, Brockmann bodies, intestine, food intake, homeostasis

## Abstract

Evidence obtained in recent years in a few species, especially rainbow trout, supports the presence in fish of nutrient sensing mechanisms. Glucosensing capacity is present in central (hypothalamus and hindbrain) and peripheral [liver, Brockmann bodies (BB, main accumulation of pancreatic endocrine cells in several fish species), and intestine] locations whereas fatty acid sensors seem to be present in hypothalamus, liver and BB. Glucose and fatty acid sensing capacities relate to food intake regulation and metabolism in fish. Hypothalamus is as a signaling integratory center in a way that detection of increased levels of nutrients result in food intake inhibition through changes in the expression of anorexigenic and orexigenic neuropeptides. Moreover, central nutrient sensing modulates functions in the periphery since they elicit changes in hepatic metabolism as well as in hormone secretion to counter-regulate changes in nutrient levels detected in the CNS. At peripheral level, the direct nutrient detection in liver has a crucial role in homeostatic control of glucose and fatty acid whereas in BB and intestine nutrient sensing is probably involved in regulation of hormone secretion from endocrine cells.

## Nutrient sensing mechanisms in fish

Since sensing and responding to fluctuations in environmental nutrient levels is a requisite for life, is not surprising that different organisms are able to detect extracellular and intracellular levels of sugars, amino acids, and lipids. The sensing of a specific nutrient may occur directly through binding of the sensed molecule to the sensor, or indirectly through detection of a related molecule that reflect nutrient abundance (Ogunnowo-Bada et al., 2014; Efeyan et al., 2015). We provide in the next sections a summary of the findings obtained in fish about glucose and fatty acid sensors.

As for the other main nutrient, amino acid, the increase in mammals in the levels of specific branched-chain amino acids (BCAA) such as leucine inhibits food intake. This process occurs through activation of amino acid sensing systems mediated by activation of target of rapamycin (mTOR) and/or inhibition of AMP-activated protein kinase (AMPK) signaling, or via activation of BCAA metabolism (Heeley and Blouet, 2016; Morrison et al., 2016). Furthermore, the deficiency in essential amino acids (including BCAA) elicits an increase in food intake through amino acid sensing systems mediated by general control nondepressable 2 and eukaryotic initiation factor 2α (Fromentin et al., 2012; Maurin et al., 2014). In fish, no studies have attempted yet to evaluate the possible presence and functioning of comparable amino acid sensing mechanisms and their relationship with food intake control. Their presence in central areas regulating food intake is however reasonable considering that most fish are carnivorous, and therefore they are strongly dependent (certainly much more than omnivorous mammals in which most studies have been carried out to date) on dietary protein/amino acid levels for functioning. The only studies available in fish demonstrated in peripheral tissues like muscle and liver the effect of changes in amino acid levels in mRNA abundance of mTOR (Seiliez et al., 2008; Wacyk et al., 2012; Tu et al., 2015; Liang et al., 2016; Xu et al., 2016).

**Figure 1:**
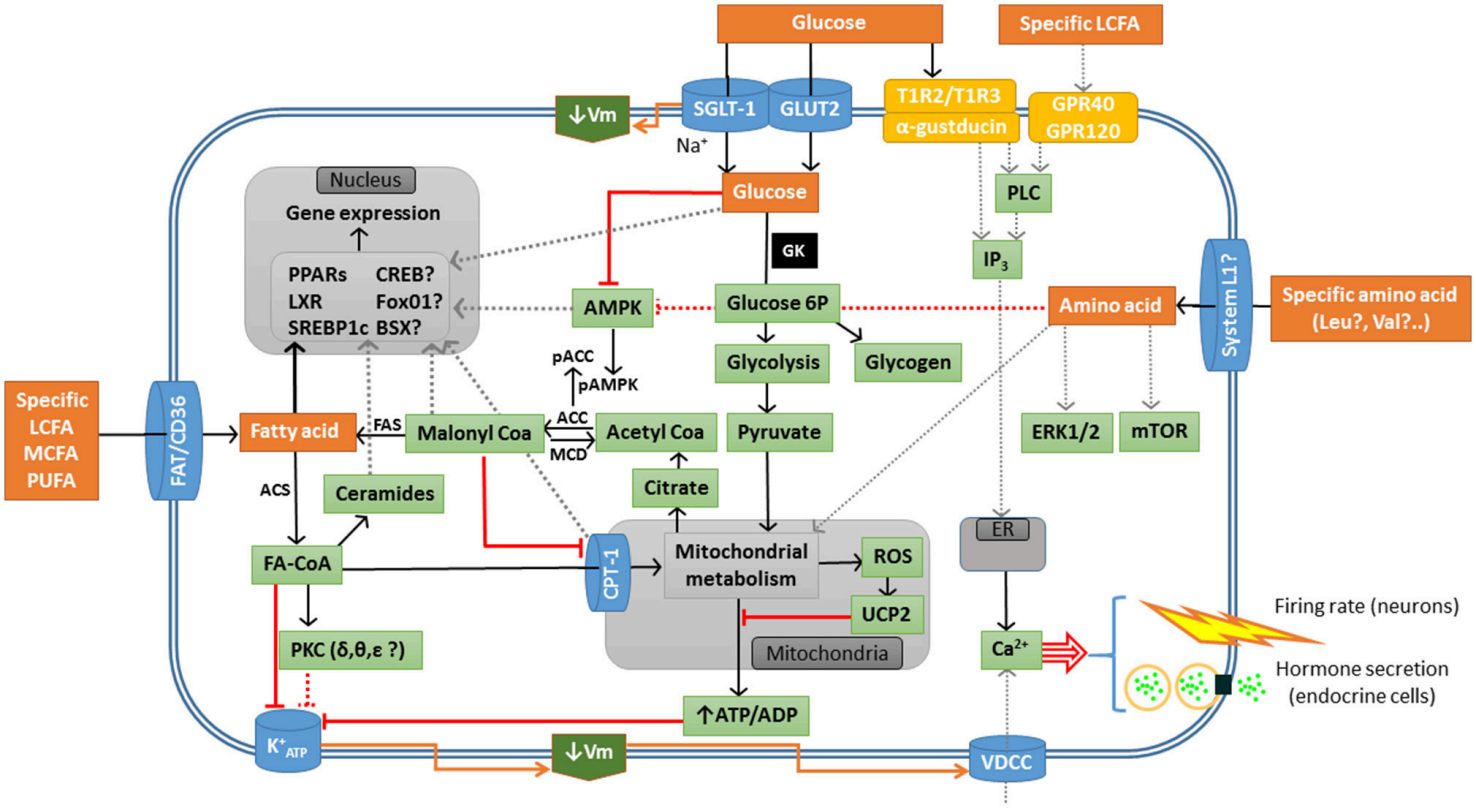
**Schematic drawing with a model of different sensing systems for glucose, fatty acid, and amino acid in sensor cells in fish**. Black line, activation; gray dotted line, hypothetical activation; red line, inhibition; red dotted line, hypothetical inhibition; ACC, Acetyl-CoA carboxylase; ACS, Acetyl-CoA synthetase; AMPK, AMP-activated protein kinase; BSX, brain homeobox transcription factor; CPT-1, carnitine palmitoyl transferase type 1; CREB, cAMP response-element binding protein; ER, endoplasmic reticulum; ERK, extracellular signal–regulated kinase; FA, fatty acid; FAS, fatty acid synthase; FAT/CD36, fatty acid translocase; FoxO1, forkhead box protein O1; ${\text{K}}_{\text{ATP}}^{+}$, inward rectifier ATP-dependent K^+^ channel; GK, glucokinase (hexokinase IV); GLUT2, facilitative glucose carrier type 2; IP_3_, inositol 1,4,5-triphosphate; GPR40. G-protein-coupled receptor 40; GPR120, G-protein-coupled receptor 120; LCFA, long-chain fatty acid; LXR, liver X receptor; MCD, malonyl-CoA decarboxylase; MCFA, medium-chain fatty acid; mTOR, target of rapamycin; PLC, phospholipase C; PPARs, peroxisome proliferator-activated receptors; SGLT-1, sodium/glucose co-transporter 1; SREBP1c, sterol regulatory element-binding protein type 1c; PKC, protein kinase C; PUFA, poly-unsaturated fatty acid; ROS, reactive oxygen species; T1R2, type 1 taste receptor subunit 2; T1R3, type 1 taste receptor subunit 3; UCP2, uncoupling protein 2; VDCC, L-type voltage-dependent calcium channel; Vm, membrane potential.

The hypothetical mechanisms involved in sensing of glucose, fatty acid, and amino acid in fish are summarized in Figure 1.

### Glucosensors

Glucosensing is the ability of specialized cells to detect changes in the levels of glucose. This ability relates to food intake control and counter-regulatory responses to changes in levels of plasma metabolites in brain areas like hypothalamus and hindbrain. In pancreatic endocrine cells and intestine it relates to hormone release whereas in liver relates to the metabolic switch between glucose utilization and production in liver. There are several glucosensing mechanisms characterized in mammals. The best known is that mediated by glucokinase (GK), as demonstrated in brain neurons, pancreatic β-cells and hepatocytes (Blouet and Schwartz, 2010; Ogunnowo-Bada et al., 2014; Efeyan et al., 2015). In this mechanism (Marty et al., 2007; Polakof et al., 2011d), glucose is taken up by glucose facilitative carrier type 2 (GLUT2), phosphorylated to glucose 6-phosphate by GK, and then metabolized through glycolysis increasing intracellular ATP/ADP ratio (Figure 1). The increased ratio induces closure of ATP-dependent inward rectified potassium channel (${\text{K}}_{\text{ATP}}^{+}$) inducing the depolarization of membrane and the entry of calcium into the cell through L-type voltage-dependent calcium channel. This entry of calcium finally results in changes in neuronal activity (brain), modulation of hormone release (endocrine cells) or changes in metabolism (liver). There is also evidence in mammals for GK-independent glucosensing mechanisms as also displayed in Figure 1 (Fioramonti et al., 2004; Marty et al., 2007; González et al., 2009; Thorens, 2012; Donovan and Watts, 2014). The expression of liver X receptor (LXR) (Mitro et al., 2007) responds to increased glucose levels eliciting a decrease in gluconeogenic capacity (Anthonisen et al., 2010; Archer et al., 2014). The sweet taste receptors (formed by type 1 taste receptor subunits (T1Rs) 2 and 3, and α-gustducin) respond to changes in glucose levels activating an intracellular signaling cascade (Ren et al., 2009; Kyriazis et al., 2014; Murovets et al., 2015; Herrera Moro Chao et al., 2016). Enhanced glucose levels induce increased expression of sodium/glucose co-transporter 1 (SGLT-1) (Díez-Sampedro et al., 2003; González et al., 2009; Thorens, 2012). The mitochondrial production of reactive oxygen species (ROS) leads to increased expression of uncoupling protein 2 (UCP2) in response to increased glucose levels (Beall et al., 2010; Diano and Horvath, 2012). These different systems might relate since, for instance, T1R3 and α-gustducin are necessary for SGLT-1 response to increased carbohydrate levels in the diet (Wauson et al., 2013).

In fish, evidence obtained in recent years support the presence of a GK-dependent glucosensing mechanism in central and peripheral areas of rainbow trout (Polakof et al., 2011d; Soengas, 2014). Indeed, in rainbow trout changes in the levels of glucose induced dietary (Polakof et al., 2008b,c), intraperitoneal (IP) (Polakof et al., 2007a, 2008a; Conde-Sieira et al., 2010a,b, 2012b; Otero-Rodiño et al., 2015), *in vitro* (Polakof et al., 2007b; Aguilar et al., 2011; Conde-Sieira et al., 2011, 2012a), or intracerebroventricular (ICV) (Polakof and Soengas, 2008) treatments resulted in changes in glucosensing mechanisms in hypothalamus and hindbrain. These include changes in GK mRNA abundance and activity, glucose and glycogen levels, GLUT2 mRNA abundance, glycolytic and glycogenic potentials, and in the activity of ${\text{K}}_{\text{ATP}}^{+}$. Besides the studies carried out in rainbow trout, a recent study provided evidence of glucose sensing properties in several hypothalamic nuclei in medaka (Hasebe et al., 2016). In peripheral tissues of rainbow trout, the presence and functioning of GK-dependent glucosensing mechanisms is supported by findings in liver (Soengas et al., 2006; Conde-Sieira et al., 2012b), Brockmann bodies (BB, main accumulation of endocrine pancreatic cells) (Polakof et al., 2007a,b, 2008b,c), and intestine (Polakof et al., 2010a; Polakof and Soengas, 2013). Interestingly, the response of glucosensing systems to glucose is more important during the day than during the night in liver but not in hypothalamus, hindbrain, and BB whose responses to hyperglycemic treatment were similar at night and day (Conde-Sieira et al., 2012a).

The presence of GK-independent glucosensing mechanisms and their response to changes in glucose levels has recently been assessed in different central and peripheral areas of rainbow trout (Polakof and Soengas, 2013; Otero-Rodiño et al., 2015, 2016a,b,c). These include hypothalamus (mitochondrial activity, sweet taste receptor, and LXR), hindbrain (SGLT-1), liver (sweet taste receptor), BB (sweet taste receptor, LXR, and mitochondrial activity), and intestine (sweet taste receptor, SGLT-1, and LXR). Furthermore, a recent study (Balasubramanian et al., 2016) also demonstrated increased mRNA abundance of T1R2 and LXR in brain of rainbow trout nutritionally programmed to cope with enhanced carbohydrate levels in the diet.

**Figure 2:**
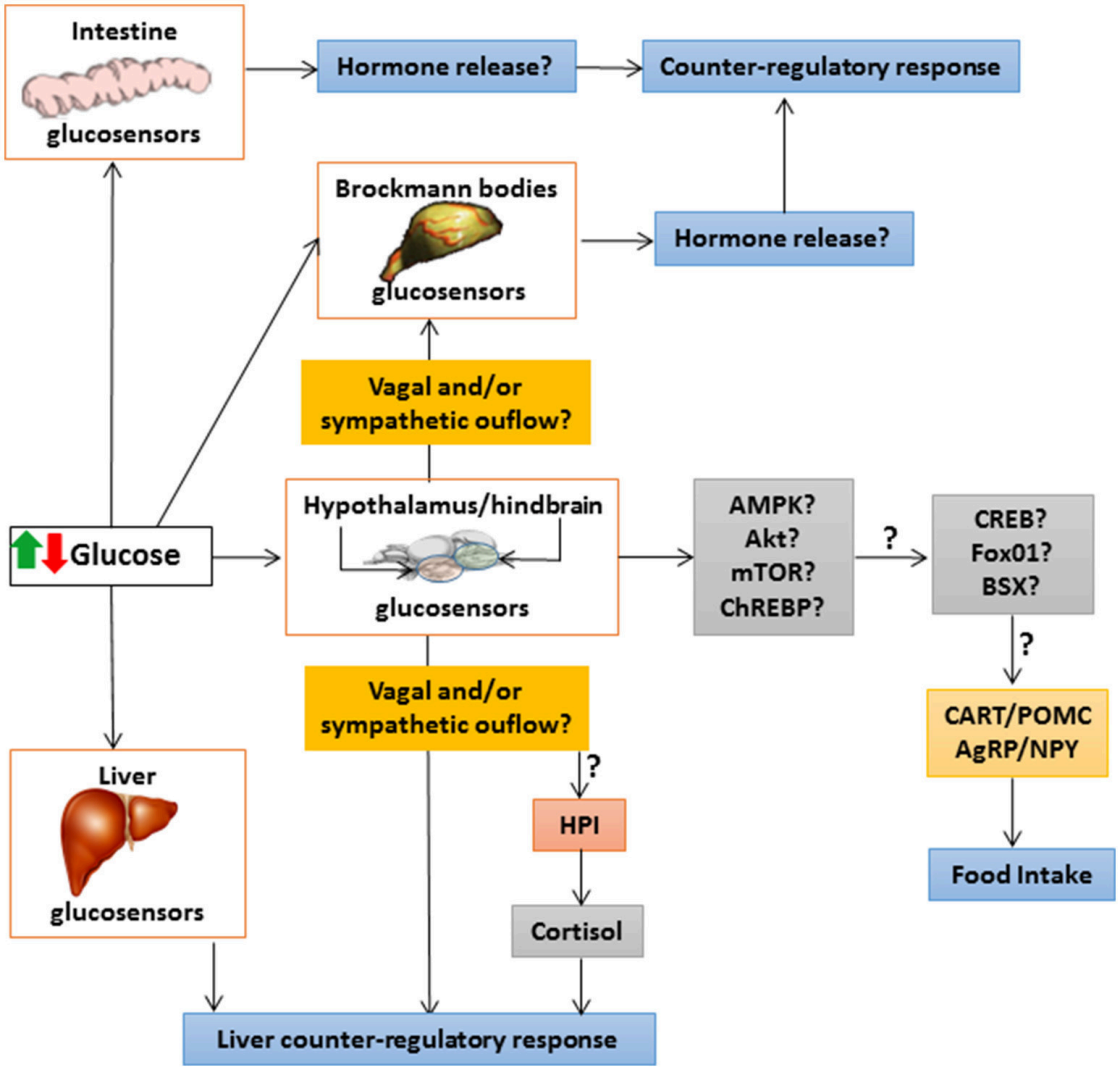
**Schematic drawing with a hypothetical model of integrative responses to an increase or decrease in glucose levels of glucosensing systems in different fish tissues**. ↑, increase; ↓, decrease; ?, unknown; AgRP, agouti-related peptide; Akt, protein kinase B; AMPK, AMP-activated protein kinase; BSX, hypothalamic homeobox transcription factor; CART, cocaine- and amphetamine-related transcript; ChREBP, carbohydrate-responsive element-binding protein; CREB, cAMP response-element binding protein; FoxO1, forkhead box protein O1; HPI, hypothalamus-pituitary-interrenal axis; mTOR, target of rapamycin; NPY, neuropeptide Y; POMC, pro-opio melanocortin.

Figure 2 summarizes the integrative responses of glucosensing systems in different fish tissues to an increase or decrease in glucose levels.

### Fatty acid sensors

In mammals fatty acid sensing systems are involved in hypothalamus and hindbrain in the detection of changes in the levels of long-chain fatty acid (LCFA) thus contributing to energy homeostasis control (Migrenne et al., 2007; Gao et al., 2013; Duca and Yue, 2014; Efeyan et al., 2015). The best known mechanism is of metabolic nature (Figure 1) in a way that a rise in LCFA levels results in increased levels of malonyl-CoA, which inhibits carnitine palmitoyl transferase-1 (CPT-1) then resulting in the inability of mitochondria to import fatty acid-CoA for oxidation (López et al., 2005, 2007). There is also evidence for the presence of alternative mechanisms in mammals (Figure 1). These include the increased binding capacity of fatty acid translocase (FAT/CD36) in response to elevated LCFA levels resulting in changes in the expression of several transcription factors (Le Foll et al., 2009). The activation of specific isoforms of protein kinase C in response to increase levels of LCFA results in the inhibition of ${\text{K}}_{\text{ATP}}^{+}$ activity (Benoit et al., 2009; Blouet and Schwartz, 2010). The activity of ${\text{K}}_{\text{ATP}}^{+}$ inhibited by increased capacity of mitochondria to produce ROS in response to increased LCFA levels (Blouet and Schwartz, 2010). Finally, the activity of lipoprotein lipase increases in response to enhanced availability of triglycerides resulting in increased levels of LCFA stimulating G-protein-coupled receptors 40 and 120 (Picard et al., 2013; Ekberg et al., 2016). These systems apparently respond to specific LCFA, such as the monounsaturated fatty acid oleate (C18:1 n-9) (López et al., 2007; Blouet and Schwartz, 2010; Duca and Yue, 2014). The ability of other classes of LCFA differing in the length of their acyl chain and/or in their degree of unsaturation to elicit the activation of these systems has been scarcely assessed to date. The available studies in mammals indicate that neither saturated fatty acids like palmitate (C16:0) nor the presence of two (such as in linoleate, C18:2 n-6) or three (such as in docosahexanoate, C22:6 n-3) double bonds activate fatty acid sensing systems (Gomez-Pinilla and Ying, 2010; Ross et al., 2010; Schwinkendorf et al., 2011; Greco et al., 2014).

Lipids are major nutrients in fish where they metabolically support many different processes (Sheridan, 1994; Tocher, 2003; Polakof et al., 2010b). Therefore, not surprisingly, many studies evaluated the effects of different dietary lipids in fish metabolism (Morash et al., 2009; Torstensen et al., 2009; Sánchez-Gurmaches et al., 2010; Figueiredo-Silva et al., 2012a,b,c; Martinez-Rubio et al., 2013). However, only recent studies provide evidence for the presence of fatty acid sensing systems in central areas of rainbow trout (Librán-Pérez et al., 2012, 2013a, 2014a,b, 2015a,b) and Senegalese sole (Conde-Sieira et al., 2015a) as well as in peripheral areas of rainbow trout (Librán-Pérez et al., 2012, 2013a,b,c, 2015c).

The treatment of rainbow trout with oleate induced responses compatible with fatty acid sensing in hypothalamus (Librán-Pérez et al., 2012, 2013a, 2014a), BB (Librán-Pérez et al., 2012, 2013a, 2015c), and liver (Librán-Pérez et al., 2013b,c, 2015c). These responses include decreased lipogenic and fatty acid oxidation capacities, reduced activity of ${\text{K}}_{\text{ATP}}^{+}$, and changes in the expression of transcription factors resultant of FAT/CD36 modulation. This response is comparable in general with that reported in mammals. Furthermore, in rainbow trout, similar responses occurred after treatment with the medium-chain fatty acid (MCFA) octanoate, and this is in contrast to mammals (Hu et al., 2011). This different behavior between fish and mammals might relate to the findings that body lipids in teleosts contain considerable amounts of MCFA (Davis et al., 1999; Trushenski, 2009) and/or MCFA oxidation in fish, at least in rainbow trout, is equally preferred compared with that of LCFA (Figueiredo-Silva et al., 2012a), in contrast with mammals (Ooyama et al., 2009). The response of fatty acid sensing systems in rainbow trout hypothalamus to increased levels of oleate or octanoate is also supported by the response of these tissues to specific inhibitors *in vitro* (Librán-Pérez et al., 2013a). Another peculiarity of fatty acid sensing systems in fish is their apparent capacity to respond to changes in the levels of polyunsaturated fatty acid (PUFA) of the n-6 and particularly n-3 series. These PUFA are very relevant for fish since their diets are particularly rich in long chain PUFA (Sargent et al., 2002) and PUFA are therefore abundant in their tissues (Mourente and Tocher, 1992; Tocher, 2003). Furthermore, the brain of marine fish is particularly rich in n-3 PUFA, mainly in α-linolenate (C18:3 n-3), eicosapentanoate (C20:5 n-3), and docosahexanoate (C22:6 n-3) (Tocher et al., 1992; Betancor et al., 2014). Conde-Sieira et al. (2015a) demonstrated that not only oleate but also α-linolenate activated fatty acid sensing systems present in the hypothalamus of Senegalese sole. This is completely different to that described in mammals (see above) and may relate to the importance of n-3 PUFA in fish. However, the capacity of PUFA to activate fatty acid sensing systems appears to be specific of certain PUFA since eicosapentanoate did not induce any significant change in fatty acid sensing systems (Conde-Sieira et al., 2015a).

Although levels of a particular fatty acid cannot be decreased, lipolysis inhibitors have been used to decrease circulating levels of all fatty acids, and this resulted in decreased activity of fatty acid sensing systems in mammals (Oh et al., 2012, 2014). A similar experimental approach in rainbow trout also resulted in the inhibition of fatty acid sensing systems in hypothalamus, BB, and liver, and these changes apparently relate to the activation of hypothalamus-pituitary-interrenal (HPI) axis (Librán-Pérez et al., 2014b, 2015d).

**Figure 3:**
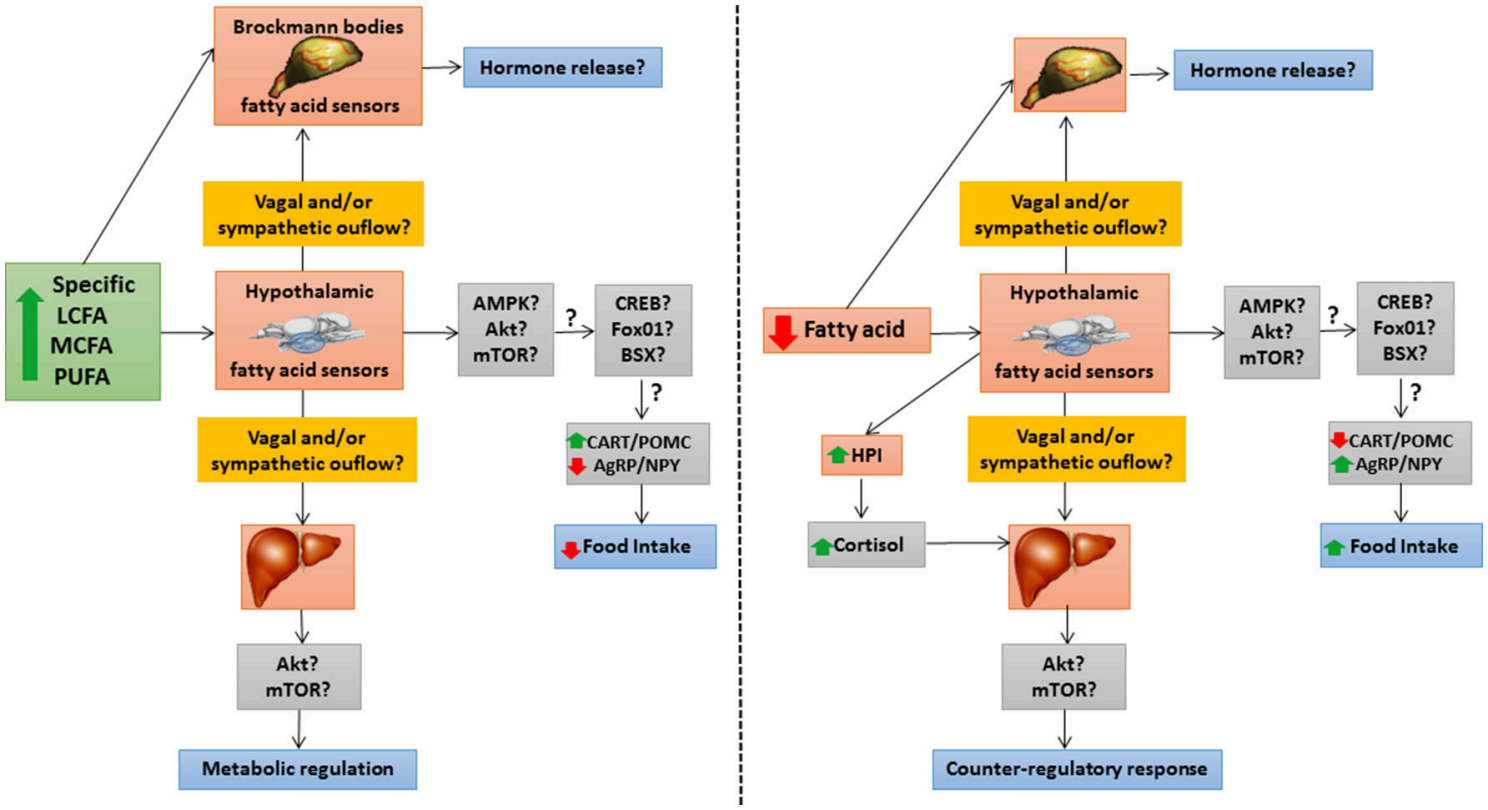
**Schematic drawing with a hypothetical model of integrative responses to an increase (left panel)** or decrease **(right panel)** in levels of specific fatty acids of fatty acid sensing systems in different fish tissues. ↑, increase; ↓, decrease; ?, unknown; AgRP, agouti-related peptide; Akt, protein kinase B; AMPK, AMP-activated protein kinase; BSX, hypothalamic homeobox transcription factor; CART, cocaine- and amphetamine-related transcript; CREB, cAMP response-element binding protein; FoxO1, forkhead box protein O1; HPI, hypothalamus-pituitary-interrenal axis; LCFA, long-chain fatty acid; MCFA, medium-chain fatty acid; mTOR, target of rapamycin; NPY, neuropeptide Y; POMC, pro-opio melanocortin; PUFA, poly-unsaturated fatty acid.

Figure 3 summarizes the integrative responses to changes in levels of specific fatty acids of fatty acid sensing systems in different fish tissues.

In summary, the evidence obtained in recent years support the presence in fish central and peripheral areas of several of the sensing mechanisms for glucose and fatty acid already characterized in mammalian models. However, these mechanisms are not exactly the same since besides responding to glucose or LCFA they also respond to other molecules such as MCFA or PUFA. Clearly, the assessment of amino acid sensing mechanism is lacking in fish, and this is of crucial importance considering that most fish species are carnivorous. Finally, there is also no information available regarding cellular mechanisms integrating information of main nutrient sensing systems into shared regulatory pathways.

## Impact of nutrient sensing on food intake regulation

In mammals nutrient detection activates directly or indirectly hypothalamic neurocircuits involved in the regulation of food intake, energy expenditure, and homeostasis (Berthoud, 2002; Morton et al., 2006, 2014; Berthoud and Morrison, 2008; Blouet and Schwartz, 2010). These circuits include two clearly defined populations of neurons mostly present in several hypothalamic nuclei including arcuate, as well as in other brain regions like hindbrain (Schwartz et al., 2000; Mobbs et al., 2005; Blouet and Schwartz, 2010; Efeyan et al., 2015). The first population responds to rises in circulating levels of glucose, fatty acid, or amino acid with the enhancement of AgRP and NPY expression. The second population responds to rises in levels of the same nutrients with enhanced co-expression of CART and POMC. Accordingly, in response to a rise in the levels of nutrients CART/POMC neurons depolarize while AgRP/NPY neurons hyperpolarize (Levin et al., 2004; Fioramonti et al., 2007). These populations also inhibit each other producing signals to higher-order neurons (Marty et al., 2007). Hypothalamic projections terminating in the hindbrain also causes a flow of efferent information to tissues involved in energy balance including liver, adipose tissue, and endocrine pancreas (Zheng and Berthoud, 2008).

In fish, NPY/AgRP and POMC/CART neurons are present in brain areas analogous to those in mammals (Cerdá-Reverter and Canosa, 2009). In addition, the expression of these neuropeptides relates to food intake control since feeding conditions change mRNA abundance of neuropeptides (Volkoff et al., 2005, 2009; Volkoff, 2006; Hoskins and Volkoff, 2012). Indeed, food deprivation decreased mRNA abundance of CART in goldfish (Volkoff and Peter, 2001), cod (Kehoe and Volkoff, 2007), and Atlantic salmon (Murashita et al., 2009) while values increased with re-feeding in channel catfish (Kobayashi et al., 2008), and post-prandial changes occurred in goldfish (Volkoff and Peter, 2001) and channel catfish (Peterson et al., 2012). As for POMC, its mRNA abundance increased post-prandially in rainbow trout (Gong and Björnsson, 2014), medaka (Chisada et al., 2014), and Atlantic halibut (Gomes et al., 2015). The mRNA levels of AgRP increased with food deprivation in hypothalamus of goldfish (Cerdá-Reverter and Peter, 2003), zebrafish (Song et al., 2003), carp (Zhong et al., 2013), and sea bass (Agulleiro et al., 2013), though not in Atlantic salmon (Murashita et al., 2009) whereas no post-feeding changes occurred in medaka (Chisada et al., 2014). AgRP mRNA levels also increased in hypothalamus of GH-transgenic carp that also displayed increased food intake (Zhong et al., 2013). The mRNA abundance of NPY decreased post-feeding in grass carp (Zhou et al., 2013), goldfish (Kehoe and Volkoff, 2007), and zebrafish (Tian et al., 2015) but responses were contradictory in orange-spotted grouper (Tang et al., 2013), rainbow trout (Gong and Björnsson, 2014), and zebrafish (Chen et al., 2016). Finally, decreased mRNA abundance of NPY occurred in food-deprived rainbow trout (Gong et al., 2016b).

### Glucosensors and regulation of food intake

In mammals, the detection of changes in glucose levels by glucosensing mechanisms results in regulatory responses, including food intake, allowing the animal to control blood glucose levels (Marty et al., 2007). Accordingly, reduced glycaemia increases food intake whereas enhanced glycaemia decreases food intake (Morton et al., 2014; Ogunnowo-Bada et al., 2014; Rogers et al., 2016).

Similar changes in food intake in response to altered glucose levels occur in fish (Polakof et al., 2011d, 2012a). Decreased food intake occurred in rainbow trout fed with a diet enriched in carbohydrates (Kaushik et al., 1989; Suárez et al., 2002; Krogdahl et al., 2004; Polakof et al., 2008b,c; Figueiredo-Silva et al., 2013). A similar response was observed after ICV or IP hyperglycaemic treatments in the same species (Ruibal et al., 2002; Polakof et al., 2007a, 2008a; Conde-Sieira et al., 2010a,b, 2012b). In contrast, increased food intake occurred in rainbow trout fed a diet with a reduced amount of carbohydrates (Sánchez-Muros et al., 1998; Capilla et al., 2003; Polakof et al., 2008b,c) or after IP or ICV hypoglycaemic treatments (Polakof et al., 2007a, 2008a; Conde-Sieira et al., 2010a,b). Comparable responses of food intake to changes in glucose levels also occurred in other fish species including goldfish (Narnaware and Peter, 2002), tilapia (Saravanan et al., 2012; Figueiredo-Silva et al., 2013), Siberian sturgeon (Gong et al., 2014) or sea bass (Castro et al., 2015).

In brain areas producing AgRP/NPY and POMC/CART histochemical studies in rainbow trout support the presence of GK (Polakof et al., 2009) suggesting a functional relationship between glucosensors and neuropeptides. However, few studies in fish described changes in the mRNA abundance of those neuropeptides in response to changes in glucose levels. The mRNA abundance of hypothalamic NPY decreased in hyperglycaemic-treated rainbow trout (Conde-Sieira et al., 2010b, 2012b; Aguilar et al., 2011; Otero-Rodiño et al., 2016a). A similar decline occurred in fish fed with a carbohydrate-enriched diet, such as in rainbow trout (Figueiredo-Silva et al., 2012c) and goldfish (Narnaware and Peter, 2002) whereas in the whole brain of gilthead sea bream no changes occurred (Babaei et al., 2017). CART mRNA levels in hypothalamus increased in response to elevated glucose levels in catfish (Subhedar et al., 2011) and rainbow trout (Conde-Sieira et al., 2010b, 2012b; Otero-Rodiño et al., 2015) or after rainbow trout were fed with a carbohydrate-enriched diet (Figueiredo-Silva et al., 2012c). Hypothalamic POMC mRNA levels increased in hyperglycaemic rainbow trout (Conde-Sieira et al., 2010b; Otero-Rodiño et al., 2015). Finally, AgRP mRNA abundance did not display changes in hypothalamus of rainbow trout after hyperglycaemic treatment (Otero-Rodiño et al., 2015, 2016a). Therefore, the mRNA abundance in glucosensing central areas (hypothalamus and hindbrain) of the four neuropeptides involved in the food intake regulation is affected by changes in glycaemia, and this is compatible with the changes observed in food intake (Polakof et al., 2008a,b).

### Fatty acid sensors and regulation of food intake

In fish fed with a lipid-enriched diet, a decrease in food intake usually takes place. This occurred for instance in rainbow trout (Peragón et al., 2000; Rasmussen et al., 2000; Gélineau et al., 2001; Forsman and Ruohonen, 2009; Figueiredo-Silva et al., 2012c; Saravanan et al., 2013), chinook salmon (Silverstein et al., 1999), polka-dot grouper (Williams et al., 2006), Senegalese sole (Bonacic et al., 2016) or grass carp (Li et al., 2016). Moreover, enhanced lipid storage is also usually associated with a reduced food intake (Shearer et al., 1997; Silverstein et al., 1999; Johansen et al., 2002, 2003). Therefore, lipid metabolism is clearly influencing food intake control in fish. Considering the relative high importance of fatty acids within the lipid pool, both in fish diets and in tissue composition, is not surprising that the available studies in fish focussed on fatty acids.

In recent studies in rainbow trout a decrease in food intake was observed after IP (Librán-Pérez et al., 2012) or ICV (Librán-Pérez et al., 2014a; Velasco et al., 2016a,b) administration of oleate or octanoate, with the effect being more important for octanoate. The effect of octanoate is specific of fish (at least rainbow trout) since in mammals treatment with this fatty acid did not affect food intake (López et al., 2007; Hu et al., 2011). Moreover, when rainbow trout fed diets containing different lipid composition, the lower food intake occurred in fish with the highest levels of fatty acid in plasma (Luo et al., 2014). This finding supports that central fatty acid sensing mechanisms mediated the lipid-induced decrease in food intake. Further support come from results obtained in rainbow trout where the decrease in food intake induced by treatment with a fatty acid synthase (FAS) inhibitor is counteracted by the simultaneous presence of an acetyl-CoA carboxylase inhibitor (Librán-Pérez et al., 2012), i.e., a response similar to that of mammals (Loftus et al., 2000; Gao and Lane, 2003; Hu et al., 2011). In Senegalese sole IP treatment with oleate, α-linolenate, or eicosapentanoate also resulted in a decrease in food intake (Conde-Sieira et al., 2015a). Furthermore, when levels of circulating fatty acid decreased through pharmacological treatment a clear increase in food intake occurred in rainbow trout (Librán-Pérez et al., 2014b).

In mammals, the activation of fatty acid sensing systems results in food intake inhibition through changes in the expression of anorexigenic and orexigenic neuropeptides (López et al., 2005; Oh et al., 2016). Accordingly, the increase in LCFA levels results in a decrease in the mRNA abundance of AgRP and NPY as well as in an increase in mRNA abundance of CART and POMC. Therefore, not surprisingly, several studies have described changes in mRNA abundance of neuropeptides in fish fed lipid-enriched diets. Feeding fish with these diets resulted in increased mRNA abundance of POMC in rainbow trout (Librán-Pérez et al., 2015b), and increased mRNA abundance of CART in rainbow trout (Figueiredo-Silva et al., 2012c; Librán-Pérez et al., 2015b) and Atlantic salmon (Hevrøy et al., 2012). Feeding diets enriched in lipids also induced a decrease in NPY mRNA abundance in grass carp (Li et al., 2010) but not in rainbow trout (Figueiredo-Silva et al., 2012c; Librán-Pérez et al., 2015b) or orange-spotted grouper (Tang et al., 2013). Finally, AgRP mRNA abundance did not change in Atlantic salmon (Hevrøy et al., 2012) but decreased in rainbow trout (Librán-Pérez et al., 2015b) fed with lipid-enriched diets.

Other available studies described the impact of treatments with specific fatty acids on mRNA abundance of orexigenic and anorexigenic neuropeptides. In rainbow trout oleate either through IP (Librán-Pérez et al., 2012), *in vitro* (Librán-Pérez et al., 2013c) or ICV (Librán-Pérez et al., 2014a; Velasco et al., 2016a,b) treatments resulted in hypothalamus in a decrease in mRNA abundance of NPY and an increase in mRNA abundance of CART and POMC. Changes observed in NPY mRNA levels after oleate treatment are comparable to those of mammals (Blouet and Schwartz, 2010). The changes displayed by neuropeptides point to an enhancement of the anorexigenic potential, which is in agreement with the effects in food intake after treatment with the same fatty acid. The treatment of rainbow trout with octanoate also resulted in decreased NPY mRNA abundance after ICV treatment (Librán-Pérez et al., 2014a), and increased mRNA abundance of CART and POMC after ICV and *in vitro* treatments (Librán-Pérez et al., 2013c, 2014a). These changes also suggest an enhancement of the anorexigenic potential in hypothalamus in response to octanoate treatment supporting the reduced food intake observed after treating the same species with octanoate (Librán-Pérez et al., 2012, 2014a). This effect of octanoate is exclusive to fish, at least rainbow trout, since in mammals octanoate does not induce any change in mRNA abundance of neuropeptides (Hu et al., 2011). In Senegalese sole, the treatment with oleate also induced a decrease in the mRNA abundance of AgRP while that of CART increased, i.e., a balance favoring an anorexigenic response (Conde-Sieira et al., 2015a). In the same species, the IP treatment with the PUFAs α-linolenate or eicosapentanoate (Conde-Sieira et al., 2015a) decreased mRNA abundance of AgRP (α-linolenate) and increased mRNA abundance of CART (α-linolenate and eicosapentanoate) thus favoring enhanced anorexigenic potential, in a way similar to the effects elicited by oleate. This was the first time in any vertebrate species in which any PUFA induced changes in hypothalamic mRNA abundance of neuropeptides involved in food intake control. Interestingly, parameters involved in fatty acid sensing changed only in the case of α-linolenate (Conde-Sieira et al., 2015a) suggesting a complex relationship between changes in fatty acid sensing and neuropeptide mRNA abundance.

In a way similar to that described above for fatty acid sensing systems and food intake responses, the decrease in rainbow trout of circulating levels of fatty acid resulted in decreased mRNA abundance of POMC and CART. This change favors enhanced orexigenic potential (Librán-Pérez et al., 2014b), i.e., the opposed response of that elicited by increased levels of fatty acids.

### Linking nutrient sensing and neuropeptide control of food intake

The mechanisms linking the function of nutrient sensing systems with changes in the expression of neuropeptides, which ultimately regulate food intake, are mostly unknown in mammals. Changes in the expression of neuropeptides might relate to modulation of forkhead box01, phosphorylated cAMP response-element binding protein, and/or brain homeobox transcription factor (Diéguez et al., 2011). The actions of these factors would result in the enhancement of CART and POMC expression and the inhibition of AgRP and NPY expression resulting in decreased food intake (López et al., 2007; Diéguez et al., 2011). However, it is not clear how these transcription factors relate to the activity of the different nutrient sensing systems. Several possibilities have been suggested in mammals (López et al., 2007; Diéguez et al., 2011; Gao et al., 2013; Morton et al., 2014) including direct action of malonyl CoA or CPT-1, indirect action through CPT-1 inhibition, modulation by AMPK, mTOR, protein kinase B (Akt), or carbohydrate-responsive element-binding protein and/or involvement of ceramides.

In fish, several recent studies carried out in rainbow trout provided evidence for several of these hypothetical mechanisms. Indeed, Librán-Pérez et al. (2015b) demonstrated that protein levels of AMPK, Akt, and mTOR increased in hypothalamus of fish fed a lipid-enriched diet. Furthermore, Gong et al. (2016a) demonstrated increased Akt protein levels in isolated hypothalamic cells incubated with leptin. Finally, Velasco et al. (2016b) also suggested the possible involvement of ceramides in the connection between activation of hypothalamic fatty acid sensing systems, neuropeptide mRNA abundance, and control of food intake. Besides these preliminary studies, there is no other evidence in fish about hypothalamic pathways related to integration of metabolic information coming from different nutrient sensor systems (glucose, fatty acids, amino acids) into a shared pathway controlling food intake via neuropeptide expression.

Food intake regulation is a complex process in which nutrient sensing systems are apparently involved in fish, in a way again comparable to that of mammals with notable differences including the capacity of several nutrients like MCFA or PUFA to modify food intake control in fish. Again, there is no information regarding the involvement of amino acid sensing systems in fish on food intake regulation, and this clearly needs assessment in the near future. Once characterized such a putative effect, the next step would be the assessment of how and why changes in those sensing systems translate into expression of anorexigenic and orexigenic neuropeptides ultimately regulating food intake. The possible mechanisms are mostly unknown, even in mamamals, and therefore it is quite probable that important differences between fish and mammals arise considering their different gastrointestinal morphology and physiology, and dietary habits.

## Impact of nutrient sensing on energy homeostasis

Nutrient sensing mechanisms in mammals are also implicated in the regulation of energy homeostasis through processes other than food intake (Levin, 2006; Blouet and Schwartz, 2010; Morton et al., 2014), such as hormone secretion and energy expenditure (Morgan et al., 2004; Pocai et al., 2005; Le Foll et al., 2009; Roh et al., 2016). The homeostatic control carried out by nutrient sensing systems occurs at both central and peripheral levels. At the central level, the brain integrates multiple metabolic inputs from the periphery as nutrients, gut-derived satiety signals and adiposity-related hormones eliciting a counter-regulatory response in peripheral tissues modulating various aspects of metabolism (Morton et al., 2014; Rogers et al., 2016). At peripheral level, nutrient sensing systems modulate energy metabolism either directly or indirectly through endocrine effectors (Marty et al., 2007; Morton et al., 2014).

### Central nutrient sensing and counter-regulation

#### Central nutrient sensing and regulation of hepatic metabolism

ICV administration of glucose or a LCFA like oleate in mammals results in a decrease of hepatic glucose production and lipogenesis (Obici et al., 2002; Morgan et al., 2004; Migrenne et al., 2011). The downstream mechanism(s) involved are presumably based on sympathetic and parasympathetic systems that provide direct innervations to liver and endocrine pancreas via the splanchnic nerve and vagus nerve, respectively (Morgan et al., 2004; Migrenne et al., 2006; Blouet and Schwartz, 2012; Roh et al., 2016).

In fish, central glucose administration affects liver metabolism. In rainbow trout ICV administration of glucose resulted in liver in decreased levels of glucose and glucose 6-phosphate, increased capacity for glycolysis and glycogenesis, and decreased capacity of glucose export into plasma (Polakof and Soengas, 2008). The presence of glucose in the brain appears to be a signal of energy abundance indicative that no production and release of glucose from liver is necessary to sustain plasma glucose levels (Polakof and Soengas, 2008). Central treatment with oleate or octanoate in rainbow trout also induced changes in several parameters related to fatty acid and glucose metabolism in liver directed to counter-regulate the elevated fatty acid levels detected in the brain (Librán-Pérez et al., 2015c). These changes in liver include increased levels of glucose and glycogen, decreased levels of fatty acids and total lipids, decreased mRNA abundance of GK and fructose 1,6-bisphosphatase as well as FAS and CPT-1 activities. The changes in glucose metabolism observed in liver are similar to those reported in mammals where ICV administration of oleate (but not octanoate) resulted in a marked decrease of hepatic glucose production via decreased glycogenolysis and glucose release (Obici et al., 2002; Morgan et al., 2004). Furthermore, the results obtained in liver metabolism were similar when comparing central (Librán-Pérez et al., 2015c) and IP (Librán-Pérez et al., 2013b) administration of fatty acid, which would indicate that sensing capacity in liver is indirect and therefore dependent on the previous sensing in brain. These changes in hepatic metabolism after central administration of glucose or fatty acid are indicative of a functional connection between central nutrient sensing and production/release of fuels from liver (Marty et al., 2007). The mechanisms involved are also likely based on sympathetic and parasympathetic systems (Morgan et al., 2004; Migrenne et al., 2006) since, at least in rainbow trout, vagus and splanchnic nerves are present in the gastrointestinal tract though it is not clear whether or not branches of those nerves arrive to the liver (Burnstock, 1959; Seth and Axelsson, 2010).

Interestingly, in rainbow the HPI axis is also likely involved in the counter-regulatory response of liver metabolism to a fall of circulating FA levels, in order to restore the normal values (Librán-Pérez et al., 2014b, 2015d), in a way comparable to that described in mammals (Oh et al., 2012, 2014).

#### Central nutrient sensing and the pancreatic counter-regulatory response

Central glucose detection is involved in mammals in the pancreatic counter-regulatory response to hypoglycaemia in order to restore normal blood glucose levels (Blouet and Schwartz, 2010). The brain, especially the hypothalamus and brain stem, receives and integrates this information to control the counter-regulatory response by modulating pancreatic insulin and glucagon secretion via the parasympathetic and sympathetic efferent nerves that innervate pancreatic α- and β-cells (Ogunnowo-Bada et al., 2014; Roh et al., 2016). This response involves suppression of insulin secretion, activation of glucagon secretion, activation of catecholamine secretion from the adrenal glands, and the activation of hepatic glucose production by the autonomic nervous system (Marty et al., 2007). Conversely, central glucose administration suppresses the counter-regulatory hormonal responses to hypoglycaemia (Roh et al., 2016). In mammals, several studies demonstrate the involvement of central glucosensors and their components in the counter-regulatory response (Miki et al., 2001; Evans et al., 2004; Sanders et al., 2004; Marty et al., 2005; McCrimmon et al., 2005). These central glucosensors can modulate not only the counter-regulatory response to hypoglycaemia in the pancreatic cells by modulating the glucagon secretion, but also the glucose-stimulated insulin secretion in the β-cells, through activation and inhibition of the sympathetic or parasympathetic branches, respectively (Thorens, 2011; Chan and Sherwin, 2012; Osundiji et al., 2012).

In fish, central administration of glucose in rainbow trout resulted in increased GK activity and expression in BB (Polakof and Soengas, 2008). This may suggest an activation of the glucosensor system in BB that could result in increased insulin levels in plasma as part of the system trying to counter-regulate the increase in plasma glucose levels elicited by ICV treatment (Polakof and Soengas, 2008). Other studies carried out in rainbow trout also support the connection between glucose levels and pancreatic function (Polakof et al., 2012a,b). Indeed, plasma insulin levels decrease and plasma glucagon levels increase in fish subjected to natural or experimental deprivation of food (Navarro and Gutiérrez, 1995). Moreover, in zebrafish exposed to high glucose levels, insulin expression was also apparently enhanced (Jurczyk et al., 2011).

Several studies in mammals suggest that not only glucose, but also fatty acid detection in central nutrient sensing areas can alter the pancreatic function through alterations of sympathetic nervous activity (Migrenne et al., 2006; Blouet and Schwartz, 2010). Central administration of lipids that do not change plasma fatty acid concentrations, induce increased glucose-induced insulin secretion counteracted by the inhibition of β-oxidation (Cruciani-Guglielmacci et al., 2004). Furthermore, oleate injection leads to increments in plasma insulin levels without altering glycaemia, suggesting that fatty acids *per se* can regulate neural control of insulin secretion (Migrenne et al., 2011).

In fish, ICV treatment with oleate or octanoate elicited several changes in BB lipid metabolism (Librán-Pérez et al., 2015c), which, in general, are different than those obtained after IP administration using the same fatty acid (Librán-Pérez et al., 2012) or to those described in mammals after ICV administration of oleate (MacDonald et al., 2008). Therefore, contrary to that observed in mammals, fatty acid sensing in BB of rainbow trout appears to be mainly direct and probably not dependent on previous central sensing. Furthermore, the action of peripheral hormones is probably influencing sensing capacity since results obtained after IP administration of fatty acid *in vivo* differed from those obtained with the same tissue *in vitro* (Librán-Pérez et al., 2013a).

### Peripheral nutrient sensing and energy homeostasis

#### Metabolic response of liver to changes in nutrient abundance

In fish, as in mammals, the regulation of glucose levels in blood depends on the balance between glucose utilization via glycolysis or glycogenesis, and glucose production via gluconeogenesis or glycogenolysis in liver. An imbalance in this regulation could be responsible of glucose intolerance in some fish species (Enes et al., 2009; Polakof et al., 2012a). This regulation relies on the differential response to variations in glycaemia of enzymes involved in hepatic metabolism. GK has been shown to be essential in fish liver for induction by glucose of key glycolytic and lipogenic enzymes and repression of genes involved in gluconeogenesis (Vaulont et al., 2000) thus acting as a glucosensor (Magnuson and Matschinsky, 2004; Polakof et al., 2011d). Thus, many fish species increased GK activity and/or expression as well as glycolytic potential in liver under hyperglycaemic conditions induced by glucose administration or by feeding diets with high contents of carbohydrates (Tranulis et al., 1996; Panserat et al., 2000; Enes et al., 2006, 2009; Conde-Sieira et al., 2010a, 2015b, 2016; Castro et al., 2016).

Changes in circulating levels of glucose also modulated other components of the GK-dependent glucosensing machinery in liver of different fish species (Hemre et al., 2002; Polakof et al., 2008b, 2011d; Enes et al., 2009). These include variations in glucose and glycogen levels, GLUT2 mRNA abundance, glycolytic and glycogenic potentials, and in the activity of ${\text{K}}_{\text{ATP}}^{+}$ occurred in the liver of hyperglycaemic rainbow trout (Conde-Sieira et al., 2010a, 2012a). Moreover, in rainbow trout fed a carbohydrate-enriched diet an up-regulation occurred in these parameters while feeding a carbohydrate-free diet resulted in a down-regulation (Polakof et al., 2008b). As for GK-independent mechanisms, experimental results obtained in fish liver indicate enhanced mitochondrial activity in response to increased levels of glucose in rainbow trout (Craig et al., 2013; Otero-Rodiño et al., 2016b). However, these responses were not reflected in other fish species such as zebrafish (Seiliez et al., 2013), red sea bream (Liang et al., 2003) or grass carp (Li et al., 2010). Furthermore, experiments *in vitro* carried out in rainbow trout did not confirm the presence of a glucosensing mechanism in liver mediated by the mitochondrial activity (Otero-Rodiño et al., 2016c). The mechanism based on sweet taste receptor appears to be operative in liver of rainbow trout since the responses obtained with this tissue *in vitro* (Otero-Rodiño et al., 2016c) are compatible with the responses described in mammalian liver (Treesukosol et al., 2011) although with some differences to those presented *in vivo* (Otero-Rodiño et al., 2016b). A glucosensor based on the hepatic LXR seems to work differentially in fish liver compared with mammals since gluconeogenesis is not inhibited by hyperglycaemia either induced by glucose administration or by feeding fish with carbohydrate-enriched diets (Panserat et al., 2001; Kirchner et al., 2008; Polakof et al., 2011d; Otero-Rodiño et al., 2016b,c). However, in other fish species such as Senegalese sole, gilthead sea bream or common carp a clear inhibition of gluconeogenesis occurred under hyperglycaemic conditions (Panserat et al., 2002b; Kamalam et al., 2013; Conde-Sieira et al., 2015b, 2016) although no studies regarding glucosensing mechanisms based on LXR are available in these species.

Other metabolic sensors regulate intermediary metabolism in mammals through control of intracellular glucose use (Polakof et al., 2012a), including AMPK (activated when the energy level in the cell is low) or mTOR (activated when the levels of nutrients increase). In fish, AMPK phosphorylation decreased in liver and mTOR phosphorylation increased in liver and muscle of rainbow trout under post-prandial conditions (Seiliez et al., 2008; Lansard et al., 2010; Polakof et al., 2011e). Furthermore, the pharmacological activation of hepatic AMPK and the inhibition of mTOR pathway induce glucose catabolism and increased gluconeogenesis besides decreased glycolysis in trout liver, respectively (Lansard et al., 2010; Polakof et al., 2011e). These findings suggest the existence in fish of a system induced by feeding carbohydrates with similar consequences on glucose metabolism as those observed in mammals (Seiliez et al., 2008; Lansard et al., 2010; Polakof et al., 2011e). Moreover, under hyperglycaemic conditions a decrease in the mRNA abundance of sirtuin-1 (another integrative nutrient sensor) is observed in liver and BB of rainbow trout (Otero-Rodiño et al., 2016b), similar to that described in mammals (Ruderman et al., 2010; Velásquez et al., 2011). Therefore, evidences exist in fish regarding the functioning of integrative energy and nutrient sensors in response to changes in the levels of a nutrient like glucose (Otero-Rodiño et al., 2016b).

Increased circulating fatty acid levels also induce metabolic changes in liver of fish as in mammals, in order to restore normal conditions. High content of lipids in the diet reduce lipogenic potential and increases β-oxidation in the liver of many fish species (Dias et al., 2004; Figueiredo-Silva et al., 2010; Borges et al., 2013; He et al., 2015; Librán-Pérez et al., 2015b; Li et al., 2016). Furthermore, dietary lipid level affects glucose metabolism inducing hyperglycaemia, and reducing glycolytic capacity and increasing gluconeogenic potential in liver, as described in several fish species like rainbow trout (Gélineau et al., 2001; Panserat et al., 2002a; Figueiredo-Silva et al., 2012a,b), other salmonids (Mazur et al., 1992; Hemre and Sandnes, 1999), grouper (Cheng et al., 2006), sunshine bass (Hutchins et al., 1998), and Senegalese sole (Borges et al., 2014). The long-term use of lipid-enriched diets in fish can compromise glucose homeostasis due to an impairment on insulin signaling and a down regulation of the Akt and mTOR pathways, as observed in rainbow trout or Senegalese sole (Panserat et al., 2002a; Figueiredo-Silva et al., 2012b; Borges et al., 2014).

Several of the putative components of fatty acid sensing mechanisms are present in fish liver (Kolditz et al., 2008; Plagnes-Juan et al., 2008; Lansard et al., 2009; Skiba-Cassy et al., 2009; Polakof et al., 2010b). Moreover, the peripheral administration of oleate or octanoate induces in rainbow trout enhanced fatty acid catabolism as well as reduced lipogenic and glycolytic potentials, suggesting a direct action of fatty acid administration on hepatic glucose and lipid metabolism (Librán-Pérez et al., 2013b). However, under *in vitro* conditions (Librán-Pérez et al., 2013c), administration of oleate or octanoate induces changes opposed of those observed *in vivo*, which indicates that fatty acid sensing capacity in liver is indirect and probably be the result of previous hypothalamic sensing. The finding that ICV treatment in rainbow trout with the same fatty acid induced changes in fatty acid sensing systems (Librán-Pérez et al., 2015c) similar to those obtained after IP treatment supports this hypothesis.

#### Nutrient sensing in BB and the modulation of hormone release

In mammals, the glucosensing mechanism based on GK present in pancreatic β-cells is involved in modulation of insulin release in response to changes in blood glucose levels (Rutter et al., 2015), which therefore constitutes an essential mechanism for the maintenance of glucose homeostasis (MacDonald et al., 2005; Polakof et al., 2011d).

Experimental evidences suggest that a glucosensor system linked to insulin secretion is present in pancreatic endocrine cells in fish. Indeed, insulin release is stimulated by glucose (Epple et al., 1987; Mommsen and Plisetskaya, 1991; Hrytsenko et al., 2008; Jurczyk et al., 2011) as well as by 2-deoxyglucose, mannose and K^+^ (Ronner and Scarpa, 1987; Ronner, 1991) and inhibited under hypoglycaemia induced by food deprivation (Navarro and Gutiérrez, 1995). These changes may relate to those observed in the pancreatic glucosensor system in fish under altered conditions of glycaemia. In rainbow trout BB these include increased GK activity and expression, GLUT2 expression, glycolytic capacity as well as glucose and glycogen levels in hyperglycaemic fish (Polakof et al., 2007a,b). In the same species, feeding fish with diets enriched in carbohydrates upregulates glucosensing response in BB whereas feeding fish with diets poor in carbohydrates resulted in a down-regulation of glucosensing response in the same tissue (Polakof et al., 2008b,c). Some GK-independent mechanisms also present in BB of rainbow trout respond to increased levels of glucose with changes in parameters related to mitochondrial activity, LXR, and sweet taste receptor both *in vivo* (Otero-Rodiño et al., 2016b) and *in vitro* (Otero-Rodiño et al., 2016c).

In mammals, lipid metabolism in the β-cell is also critical for the normal regulation of insulin secretion (MacDonald et al., 2008) and fatty acids directly regulate insulin release from pancreatic β-cells (Nolan et al., 2006). In fish, the available experimental results also demonstrate enhanced insulin release in response to increase levels of fatty acid (Barma et al., 2006). Moreover, insulin treatment in rainbow trout enhances the potential of lipogenesis and decreases the potential of fatty acid oxidation in several tissues (Plagnes-Juan et al., 2008; Lansard et al., 2010; Polakof et al., 2010b, 2011d; Caruso and Sheridan, 2011). In rainbow trout, the decreased mRNA levels of FAS and CPT1c in BB after treatment with oleate or octanoate (Librán-Pérez et al., 2012) suggest that components of putative fatty acid sensing systems respond in BB to increased fatty acid levels. This response could modulate insulin secretion from this tissue, as reported in mammals (Keane and Newsholme, 2014), with the main difference that in fish fatty acid sensing systems are also responsive to a MCFA like octanoate. This mechanism appear to be mainly the result of a direct action of fatty acid in β-cells (Librán-Pérez et al., 2013a) though an indirect action by previous hypothalamic sensing mediated by vagal and/or splanchnic outflow cannot be discarded (Librán-Pérez et al., 2015c).

#### Glucosensing capacity in gut

The gastrointestinal tract in mammals has an important role in the complex signaling network that controls food intake, metabolism and energy homeostasis since it releases several energy-related gastrointestinal hormones that send nutritional information to the control areas in the brain through afferent nerves (Schwartz et al., 2000; Roh et al., 2016). Accordingly, the presence of nutrient sensing mechanisms have been proposed in mammalian enteroendocrine cells (Miguel-Aliaga, 2012) and enterocytes (Pfannkuche and Gäbel, 2009). Glucose can be sensed in the gastrointestinal tract by mechanisms dependent on sweet taste receptors and gustducin, which are activated by glucose leading to the release of glucagon-like peptide 1 (GLP-1) and gastric inhibitory polypeptide (Kokrashvili et al., 2009; Miguel-Aliaga, 2012). Other glucosensing mechanisms controlling hormonal release in mammalian gastrointestinal tract involve electrogenic or metabolic processes mediated by SGLT-1 and GLUT2/GK (Miguel-Aliaga, 2012).

In fish intestine, histochemical studies evidence the presence of components of different glucosensing systems (SGLT-1 and GK) in enterocytes and enteroendocrine cells of rainbow trout (Polakof et al., 2010a). Furthermore, molecular evidence also pointed to the presence in fish intestine of glucosensing mechanisms involving components of metabolic (GK/GLUT2), electrogenic (SGLT-1), nuclear (LXR) and sweet taste receptor systems (Ishimaru et al., 2005; Geurden et al., 2007; Hashiguchi et al., 2007; Kirchner et al., 2008; Cruz-García et al., 2009; Polakof et al., 2010a). However, only few studies characterized the response of these systems in intestine to increased levels of glucose. In black bullhead enterocytes enhanced glucose uptake through SGLT-1 occurred in fish fed a diet rich in carbohydrates (Soengas and Moon, 1998) whereas in zebrafish GLUT2 mRNA abundance in intestine changed in parallel with changes in glucose levels (Castillo et al., 2009). In rainbow trout, increased glycogen levels, GK activity, glycolytic capacity, and transcript levels of GK, SGLT-1, and LXR, as well as decreased transcript levels of T1R and gustducin occurred in intestine of hyperglycemic trout (Polakof et al., 2010a; Polakof and Soengas, 2013). These systems seem to operate in fish in a different way compared with other vertebrate species (Polakof and Soengas, 2013) but certainly appear to be functional, and thus presumably involved in fish gastrointestinal physiology, especially through production and release of gastrointestinal hormones.

#### Possible glucosensing capacity in head kidney and its role on cortisol release

One study using head kidney perifused cultures in rainbow trout demonstrated that in the presence of ACTH, cortisol release increased in parallel with the increase of glucose in the medium (Conde-Sieira et al., 2013). These changes could relate to the presence of a glucosensing system in putative interrenal cells in head kidney that would respond to glucose levels in a way similar to that of pancreatic β-cells for insulin release. Accordingly, immunohistochemical studies indicate the presence of GK protein in interrenal cells and SGLT-1 protein in both interrenal and chromaffin cells of rainbow trout (Conde-Sieira et al., 2013). However, metabolite levels and enzymes activities involved in glucosensing mechanisms did not show a clear response to changes in circulating glucose levels in head kidney of rainbow trout, probably due to the high cellular heterogeneity of the tissue assessed (Conde-Sieira et al., 2013). A further study in rainbow trout (Gesto et al., 2014) supports that cortisol release under stress conditions in rainbow trout might relate to hyperglycemia previously elicited by catecholamine action.

As a whole, the nutrient sensing systems characterized in fish are involved in the regulation of energy homeostasis through mechanisms other than regulation of food intake. The evidence obtained in recent years pointed to a role of these systems in counter-regulatory mechanisms as well as in the regulation of hormone release, though the evidence is preliminary in some cases.

## Endocrine modulation of nutrient sensing

Several hormones modulate the response of nutrient sensing systems in mammals to changes in the levels of nutrients. These hormones provide information about homeostasis, status of energy stores, and the presence of food and its composition in the gastrointestinal tract. These include ghrelin, insulin, leptin, cholecystokinin (CCK), GLP-1, adiponectins, cannabinoids, and glucocorticoids (Diéguez et al., 2009; Blouet and Schwartz, 2010; Morton et al., 2014).

Results obtained in recent years in fish provide evidence for the modulatory role of several of these hormones in the activity of nutrient sensing systems as well as in the mRNA abundance of neuropeptides related to the control of food intake. Moreover, several of these hormones modulate peripheral nutrient sensing systems.

As in other vertebrates, insulin administration modifies glucose and lipid metabolism in fish, by enhancing the glucose uptake in liver and muscle, increasing hepatic glycolytic and lipogenic potentials, and depressing gluconeogenesis and fatty acid oxidation (Mommsen and Plisetskaya, 1991; Plagnes-Juan et al., 2008; Jin et al., 2014). The effects on lipid metabolism depend on the dose of insulin administered as well as the feeding status of fish (Polakof et al., 2010b, 2011f). Insulin is present and synthesized in fish brain (Caruso et al., 2008) where insulin receptors are also present (Gutiérrez and Plisetskaya, 1994; Leibush et al., 1996). Insulin treatment resulted in contradictory effects in food intake in fish. In rainbow trout IP administration of insulin inhibited (Librán-Pérez et al., 2015a) or activated (Polakof et al., 2008a; Conde-Sieira et al., 2010b) food intake whereas ICV treatment with insulin inhibited food intake in rainbow trout (Soengas and Aldegunde, 2004) but not in catfish (Silverstein and Plisetskaya, 2000). The putative anorectic effects of insulin would be in agreement with the increased anorexigenic potential elicited by insulin treatment as demonstrated increased mRNA abundance of CART in rainbow trout (Librán-Pérez et al., 2015a) and catfish (Subhedar et al., 2011) as well as decreased NPY mRNA abundance in rainbow trout (Librán-Pérez et al., 2015a). As for insulin capacity to modulate the activity of nutrient sensing systems, its administration in rainbow trout inhibits glucosensing response in hypothalamus, hindbrain, BB, and intestine (Polakof et al., 2007a, 2008a, 2010b; Conde-Sieira et al., 2010b). As for fatty acid sensing systems, no clear effects of insulin treatment were observed in rainbow trout hypothalamus (Librán-Pérez et al., 2015a), in contrast to mammals (Duca and Yue, 2014). However, in liver and BB insulin treatment potentiates the effect of oleate and octanoate on fatty acid sensing systems (Librán-Pérez et al., 2015a).

Leptin treatment is usually anorectic in fish as demonstrated studies in rainbow trout (Murashita et al., 2008; Kling et al., 2009; Aguilar et al., 2010; Gong et al., 2016a), goldfish (Volkoff et al., 2003; de Pedro et al., 2006; Vivas et al., 2011) and striped bass (Won et al., 2012). This anorectic effect occurred in parallel with changes in the expression of neuropeptides generally indicating an enhanced anorexigenic potential. Thus, leptin treatment induced a decrease in NPY mRNA levels in hypothalamus of rainbow trout (Murashita et al., 2008; Aguilar et al., 2011), hypothalamus and telencephalon of goldfish (Volkoff et al., 2003), and in whole brain in grass carp (Li et al., 2010). POMC mRNA abundance increased in response to leptin treatment in rainbow trout (Murashita et al., 2008; Aguilar et al., 2011; Gong et al., 2016a). Leptin treatment also increased CART mRNA levels in hypothalamus of goldfish (Volkoff and Peter, 2001), catfish (Subhedar et al., 2011), and rainbow trout (Murashita et al., 2008; Aguilar et al., 2011; Gong et al., 2016a). Furthermore, leptin receptor knockout for medaka displayed (compared with the wild type) a higher food intake, as well as decreased POMC mRNA abundance, and increased NPY and AgRP mRNA abundance (Chisada et al., 2014) whereas zebrafish knockout for leptin displayed changes in mRNA abundance of genes related to glucose but not to lipid metabolism (Michel et al., 2016). The anorectic effects of leptin could relate, at least in part, to the activation of nutrient sensing systems. In fact, leptin treatment clearly activates central glucosensing systems in rainbow trout (Aguilar et al., 2010, 2011). There is little evidence for the action of leptin on nutrient sensing systems in peripheral tissues of fish. The only available study showed that ICV leptin treatment in rainbow trout did not affect liver glucosensing capacity, although an increased glycogenolytic potential possibly mediated by the activation of the sympathetic nervous system occurred in rainbow trout liver (Aguilar et al., 2010).

Few studies have assessed the effects of GLP-1 on food intake in fish to date. GLP-1 treatment resulted in an inhibition of food intake in catfish (Silverstein et al., 2001) and coho salmon (White et al., 2016) but not in channel catfish (Schroeter et al., 2015). In rainbow trout GLP-1 treatment (Polakof et al., 2011b) elicited in hypothalamus and hindbrain the activation of glucosensing systems with increased mRNA abundance of CART and POMC, and decreased mRNA abundance of NPY, i.e., changes clearly indicative of enhanced anorexigenic potential. In the same species, GLP-1 IP treatment also resulted in the activation of GK-mediated glucosensing mechanism in liver (Polakof et al., 2011b).

Treatments with CCK produce anorectic responses in fish as demonstrated in rainbow trout (Gélineau and Boujard, 2001; Jönsson et al., 2006), coho salmon (White et al., 2016), goldfish (Himick and Peter, 1994; Kang et al., 2010), catfish (Silverstein and Plisetskaya, 2000), sea bass (Rubio et al., 2008), and winter flounder (MacDonald and Volkoff, 2009). Furthermore, CCK treatment in rainbow trout activated glucosensing capacity in hypothalamus and hindbrain (Polakof et al., 2011a), and this is accompanied by decreased NPY mRNA levels in hindbrain and hypothalamus, thus supporting increased anorexigenic potential. In liver of rainbow trout IP administration of CCK also activated glucosensing capacity (Polakof et al., 2011a).

The effects of ghrelin treatment on food intake in fish are controversial. Increases were noted in goldfish (Miura et al., 2006), brown trout (Tinoco et al., 2014), rainbow trout (Velasco et al., 2016a,b), striped sea bass (Picha et al., 2009) or cavefish (Penney and Volkoff, 2014) whereas decreases occurred in rainbow trout (Jönsson et al., 2010), channel catfish (Schroeter et al., 2015), and tilapia (Peddu et al., 2009). In rainbow trout ghrelin treatment activates central glucosensing systems (Polakof et al., 2011c), an effect opposed of that in mammals (Wang et al., 2008). In contrast ghrelin treatment induces an inhibition of fatty acid sensing systems in rainbow trout hypothalamus and hindbrain (Velasco et al., 2016a,b) in a way similar to that described in mammals, and these changes agree with those of mRNA abundance of neuropeptides that decreased for POMC/CART and increased for AgRP/NPY. Increased mRNA abundance of NPY occurred in hypothalamus of ghrelin-treated goldfish (Miura et al., 2006). Central ghrelin treatment also modulates indirectly hepatic liver metabolism resulting in increased potential for lipogenesis and decreased potential for fatty acid oxidation, as indicative of inhibition of fatty acid sensing (Velasco et al., 2016c).

A reduction in food intake is a typical response to stress in fish, and at least part of this response might depend on changes in the ability of stress to alter nutrient sensing systems regulating food intake. A readjustment in the activity of hypothalamic glucosensing mechanisms occurred in stressed rainbow trout (Conde-Sieira et al., 2010a; Otero-Rodiño et al., 2015). This effect might relate to any of the components of the HPI axis such as corticotropin releasing factor (CRF), which is involved in the effects of stress on food intake in mammals (Evans et al., 2004; McCrimmon et al., 2006). Accordingly, the treatment of rainbow trout hypothalamus with CRF altered functioning of glucosensing mechanisms (Conde-Sieira et al., 2011) in a way similar to that observed under stress conditions (Conde-Sieira et al., 2010a).

Finally, melatonin is mainly involved in fish in the timing of rhythmic events, but also in growth, endocrine function, and metabolism (Falcón et al., 2010). In rainbow trout, melatonin *in vitro* treatment in hypothalamic tissue activated glucosensing mechanisms and elicited a response in the expression of neuropeptides compatible with an enhancement of orexigenic potential (Conde-Sieira et al., 2012a). In contrast, in liver a clear down-regulation of glucosensing potential occurred in response to melatonin treatment (Conde-Sieira et al., 2012b). This differential tissue response to melatonin treatment might relate to the day-night differences in glucosensing capacity observed in liver of rainbow trout (Conde-Sieira et al., 2012b).

In summary, several hormones involved in the regulation of energy homeostasis are involved in the modulation of glucose and fatty acid sensing systems in fish. Despite most studies were carried out with glucosensing systems, few with fatty acid sensing systems and none with putative amino acid sensing systems, a preliminary conclusion can be obtained in a way that anorexigenic/anabolic hormones demonstrated to activate nutrient sensing systems whereas orexigenic/catabolic hormones inhibit them. There are differences in the direction and magnitude of the responses compared with the mammalian model, which among other reasons might relate to the high degree of hormone variants present in fish (as a result of their additional genome duplication), and/or to the clear difference in dietary habits between both models.

## Conclusions

**Figure 4:**
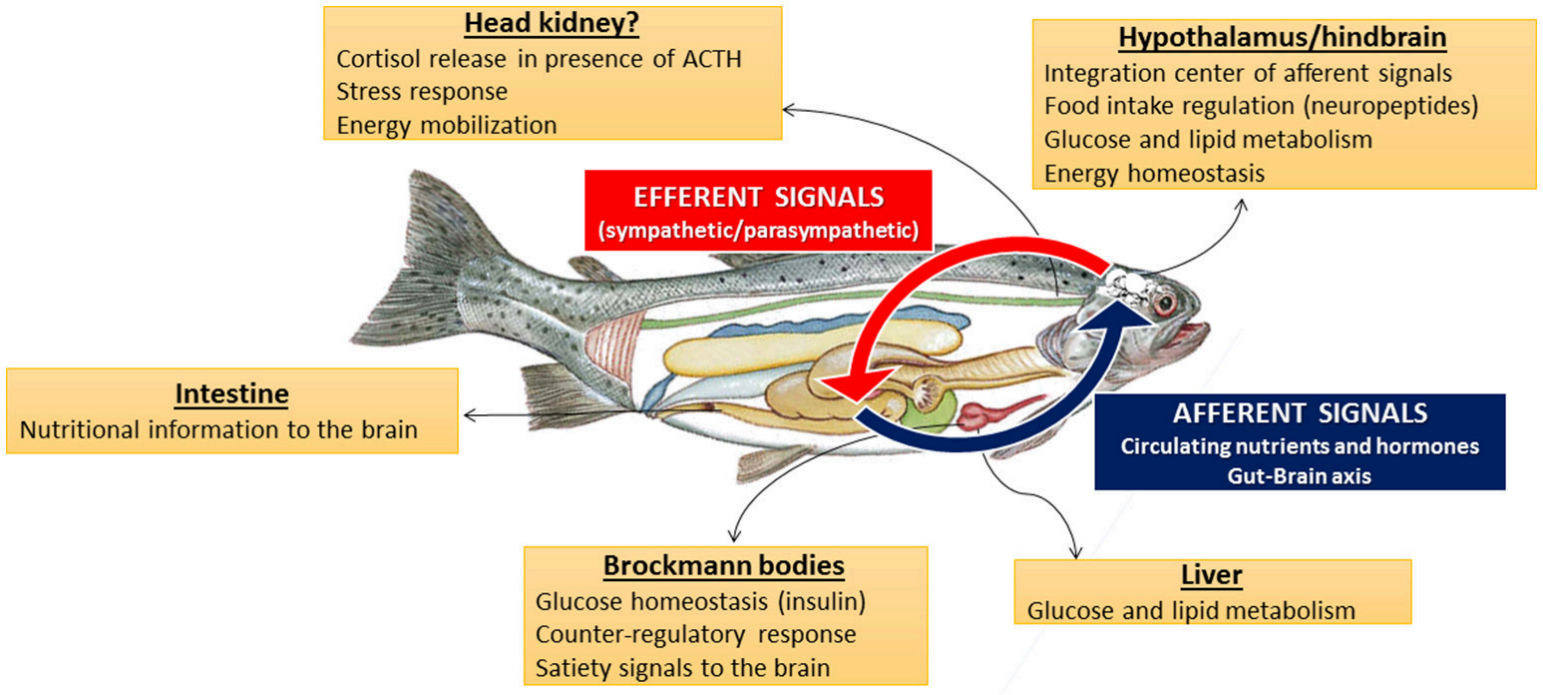
**Schematic drawing summarizing functions of nutrient sensing systems in central and peripheral tissues of fish**.

Research carried out in recent years provided information for the presence and functioning of putative nutrient sensing systems either in peripheral or central areas of the few fish species assessed to date regarding this issue, mainly rainbow trout, as summarized in Figure 4.

The main role of these systems is to participate in the control of homeostasis through modulation of feeding behavior or other processes such as energy expenditure or hormone secretion. The known mechanisms are comparable to those of mammals in several aspects but clear differences arise in others, such as the fish capacity of detecting changes in circulating levels of MCFA or PUFA. These differences between fish and mammals might relate to at least three different reasons, among others. A first reason might relate to the large importance of amino acids for metabolic purposes in fish, not only in carnivorous but also in herbivorous and omnivorous species. A second reason may be due to the high variety of dietary fish habits resulting in large differences in gastrointestinal morphology and function. A third reason may rely on the existence in fish of multiple gene variants in neuropeptides, hormones, and metabolic effectors resulting from the additional gene duplication of actinopterygians. The assessment of these topics, together with the possible presence and functioning of amino acid sensing systems in fish, as well as the elucidation of signaling pathways linking activity of sensors with the effectors controlling homeostasis, such as expression of neuropeptides controlling food intake, hormone secretion or metabolic changes, are open questions demanding further research in the near future.

## Author contributions

Both authors designed the paper, wrote and approved the final version of the manuscript.

## Funding

The authors acknowledge grants from Spanish Agencia Estatal de Investigación and European Fund for Regional Development (AGL2016-74857-C3-R and FEDER) to JS. MC was recipient of a postdoctoral fellowship from Xunta de Galicia.

### Conflict of interest statement

The authors declare that the research was conducted in the absence of any commercial or financial relationships that could be construed as a potential conflict of interest.
